# The Effect of Vocal Intonation Therapy on Vocal Dysfunction in Patients With Cervical Spinal Cord Injury: A Randomized Control Trial

**DOI:** 10.3389/fnins.2022.860127

**Published:** 2022-06-10

**Authors:** Xiaoying Zhang, Yi-Chuan Song, De-Gang Yang, Hong-Wei Liu, Song-Huai Liu, Xiao-Bing Li, Jian-Jun Li

**Affiliations:** ^1^School of Rehabilitation Medicine, Capital Medical University, Beijing, China; ^2^China Rehabilitation Science Institute, Beijing, China; ^3^Beijing Key Laboratory of Neural Injury and Rehabilitation, Beijing, China; ^4^Center of Neural Injury and Repair, Beijing Institute for Brain Disorders, Beijing, China; ^5^Music Therapy Center, China Rehabilitation Research Center, Beijing, China; ^6^Laboratory of Music Artificial Intelligence, Central Conservatory of Music, Beijing, China; ^7^Department of Spinal and Neural Functional Reconstruction, China Rehabilitation Research Center, Beijing, China

**Keywords:** cervical spinal cord injury, vocal intonation therapy, vocal dysfunction, music therapy, vocal quality

## Abstract

In this study, the vocal intonation therapy (VIT) was compared with the standard respiratory therapy for people suffering from respiratory dysfunction as a result of cervical spinal cord injury (CSCI) to observe its effect on vocal quality. Thirty patients with vocal dysfunction after CSCI with the injury time of more than 3 months were screened for inclusion in the trial, and 18 patients completed the 12-weeks, each participant had 60 sessions in total in the clinical trial. All patients were allocated to the intervention group or the control group. The intervention group received VIT training and the control group received respiratory phonation therapy. Both groups were trained by professional therapists, and the training time was 30 min/day, 5 days/week, for 60 sessions for each group in a total of 12 weeks. In the Baseline (T0), mid-intervention period (after 6 weeks, T1), and after intervention (after 12 weeks, T2), the vocal quality of the two groups of patients was tested with a computer-aided real-time audio analyzer 2.1.6 (Adobe Systems, United States) for Sing-SPL (*p* < 0.0001), Speech-SPL (*p* < 0.0001), SNL (*p* < 0.0001), and F0 (*p* < 0.0001) of the intervention group were significantly improved compared with the control group. In comparing the spectrometry analysis of vocal quality for the 2 groups of participants, there was a significant difference in the results of Sing-SPL and Speech-SPL acoustic analysis in the intervention group of patients at T2 (after 12 weeks) compared to the control group. Vocal intonation therapy—music therapy can improve the speech sound quality of cervical CSCI patients and provide CSCI patients with a practical, highly operable treatment that has both functional training effects and can bring a pleasant experience that can be promoted in the medical field. This study was approved by the Ethics Committee of China Rehabilitation Research Center (CRRC) (approval No. 2019-83-1) on May 20th, 2019. It was registered with the National Health Security Information Platform, medical research registration, and filing information system (Registration No. MR-11-21-011802) on January 28th, 2021.

## Introduction

Cervical spinal cord injury (CSCI) is a critical injury that often entails disability. Dyspnea may occur in patients with upper CSCI or quadriplegia if the diaphragm and intercostal muscles are paralyzed, often resulting in severe restrictive ventilatory impairment or medical social burden (Koda et al., 2021). Vocal dysfunction due to respiratory disorders following CSCI often presents difficulties in articulation and vocal endurance. Therefore, CSCI often causes a negative effect on the quality of patients’ vocalization (Williams et al., 2020). Presently, the prevalence of respiratory dysfunction caused by CSCI and the positive correlation between it and voice output has been proposed (Mesbah et al., 2021); however, most studies have focused on the dysfunction of the respiratory system caused by CSCI (Lemos et al., 2020), and not many studies have been conducted on the vocalization function and voice quality after CSCI.

After CSCI, the motor function of the phrenic nerve innervation is affected, respiratory function is impaired, and pronunciation quality is also affected to a certain extent. The most important manifestations are patients’ decreased volume in daily communication, the difficulty in adhering to the oral expression of long sentences, and the increase of inspiratory time (Wadsworth et al., 2012). In recent years, some studies have explored breathing and voice function after CSCI and found that respiratory dysfunction after CSCI usually results in overcompensation of lung function to deal with decreases in expiratory muscle compliance and increase speech loudness for conversation (Lu et al., 2008; Tamplin et al., 2011; MacBean et al., 2017). Some studies have demonstrated that healthy individuals only require 20% of their respiratory lung capacity for natural volume pronunciation or vocalization (Tamplin et al., 2013). In comparison, patients with respiratory dysfunction after CSCI can use 30–50% of their lung capacity depending on the extent of the injury (Hixon et al., 1973). In conclusion, it is common for CSCI patients to overcompensate for lung function to ensure the normal speech volume. Moreover, during the emergency medical treatment of CSCI patients, laryngeal dysfunction caused by intubation and tracheotomy can also lead to a mild vocal cord movement disorder, and in severe cases, vocal cord polyps, vocal nodules, or complete vocal cord paralysis (Watson and Hixon, 2001).

Some studies conducted auditory perception assessment and analysis on patients with vocalization dysfunction after CSCI and concluded that their speech features were of a reduced volume, mostly short sentences, increased inhalation time (Gadomski et al., 2021; OSCIS investigators et al., 2021), and deviations in articulation, articulation accuracy, and speech quality (Tamplin et al., 2014; Scheuren et al., 2021). Although most CSCI patients can maintain the volume required for a normal conversation in a quiet room, patients may have difficulty raising the volume in the presence of other noises (Berlowitz et al., 2016). These decreases in volume and length are directly caused by impaired respiratory function (Clini et al., 2018). Studies have also concluded that CSCI patients can significantly feel more drastic volume problems than healthy controls (Oraee et al., 2021). Some scholars have used singing to perform breathing training for quadriplegic patients, and found that singing activates accessory respiratory muscles (as measured by surface electromyography) and stimulates greater respiratory function than speaking (Tamplin et al., 2011). A follow up study in this area with 24 patients, found improvements in speech loudness (SPL) and maximum phonation length, supported by improved respiratory function following a therapeutic singing intervention (Tamplin et al., 2013). The Perceptual Voice Profile (PVP) and Voice Handicap Index (VHI), both are subjective rating scale, are used to assess vocal function scores (Crispiatico et al., 2021). The results showed that CSCI patients had low voice quality, insufficient volume in long space, insufficient breath during speech, and common social disorder problems of varying degrees in social situations.

Since singing and speaking share the same neural network, in recent years, an increasing number of studies have focused on the treatment of speech abnormalities caused by functional disorders of the nervous system (Jasmin et al., 2016). Vocal vocalization therapy (VIT) (Thaut and Hoemberg, 2014) uses vocal music training to practice vocal function control problems caused by structural, neurological, physiological, psychological, or functional abnormalities of vocal organs (Strohl et al., 2020). VIT directly stimulates the muscles associated with breathing, vocalization, articulation, and resonance and requires more sound control (Scheuren et al., 2021) and intensity (Rodrigues et al., 2021) than speech. Furthermore, VIT training can enhance respiratory muscle strength (Tamplin et al., 2014; Zhang et al., 2021). VIT training used in clinical practice resembles open voice exercises used by vocal teachers or choral conductors, addressing issues related to vocal function control, such as breath control, tone, pitch, timbre, and strength issues. VIT starts with a melody and uses various vowel and consonant vocalization combinations to provide practical and effective non-invasive treatment for patients with vocal dysfunction after spinal cord injury. Under the guidance of the music therapist, patients gradually adjust the vocal apparatus during the vocalization process and then further combine with the complete singing, conducive to improving the vocalization function and voice quality. In this study, VIT was used to intervene in patients with vocal dysfunction after cervical spinal cord injury and compared patients who received routine breathing pronunciation training. This process gauged whether VIT can achieve faster and more effective functional recovery than physical therapy to find a faster and effective treatment for patients with CSCI.

## Participants and Methods

This study was approved by the Ethics Committee of China Rehabilitation Research Center (CRRC) (approval No. 2019-83-1) on May 20th, 2019, and informed consent was obtained from the participants, relatives, or guardians before commencing the study. The study trial was registered with the National Health Security Information Platform, medical research registration, and filing information system (Registration No. MR-11-21-011802) on January 28th, 2021.

### Participants

Eighteen patients with spinal cord injury, including complete spinal cord injury and incomplete spinal cord injury, who was hospitalized in CRRC from January 2021 to December 2021, were recruited. Inclusion criteria: (1) classified as spinal cord injury class A and class B by the American spinal cord injury association (ASIA) (American Spinal Cord Injury Association [ASIA]); (2) The course of the disease should have been at least 3 months (inclusive); (3) Aged between 18 and 75; (4) Moderate or above respiratory dysfunction and phonation disorder after cervical spinal cord injury, voice volume in normal conversation is lower than 40 decibels; (5) no tracheotomy or tracheotomy has healed; (6) Can tolerate seat training for more than 15 min at a time without postural hypotension; (7) No previous music learning experience; (8) native Mandarin Chinese; (9) Patients and their families were informed and consented to this study. Exclusion criteria: (1) a history of severe speech disorder, a history of mental disorder, or a history of severe respiratory disease before injury; (2) Severe cognitive dysfunction, MMSE score < 17 (illiteracy) or < 20 (primary school); (3) tracheotomy, vocal cord damage, or posterior damage; (4) epilepsy, malignant arrhythmia, or other serious physical diseases. Criteria for withdrawal and termination: The study can be promptly terminated if the patient’s condition changes or discharged or voluntarily quit the study. The general information of patients is as follows (Table 1).

**TABLE 1:** Participants’ characteristics in this study.

	Intervention group	Control group	*T*	*p*
Total number	9	9		>0.05
Gender				
Male	7	8		>0.05
Female	2	1		>0.05
Age	38.60 ± 17.89	34.78 ± 11.13	0.6186	>0.05
Months since injury	4.20 ± 4.0	5.64 ± 4.08	0.0109	>0.05
Height (cm)	172.00 ± 11.00	166.10 ± 9.00	1.25	>0.05
Weight (kg)	63.20 ± 9.90	63.90 ± 17.92	0.9919	>0.05
BMI	21.37 ± 3.03	22.91 ± 5.24	0.0223	>0.05
AISA classification				
ASIA A	6	5		>0.05
ASIA B	3	4		>0.05
Injury level grading				
C4	5	6		>0.05
C5	4	3		>0.05

*Data were expressed as the number in total number, gender, age, months since injury (time), height (cm), weight (kg), body mass index (BMI), AISA classification, and injury level grading. Other data were expressed as the mean ± SD and analyzed by paired t-test. Intervention group: vocal intonation therapy (VIT) group; Control group: respiratory phonation training. ASIA, American Spinal Injury Association.*

### Research Design

Since this study was a randomized controlled trial, *n* = Z2⋅σ2/d2 was calculated according to the sample size formula, where n was the minimum sample size. Z is the confidence interval, usually 90%; Sigma is the standard deviation; D is the sampling error range; usually, 0.5 and the minimum sample size was thus calculated. Therefore, this study met the requirement of a minimum sample size (n ≈ 18) and was divided into two groups. Computer-generated sequences (Excel 2013, US, Washington, Seattle, Microsoft Office) randomly divided patients into two groups: vocal intonation therapy group (*n* = 9) and respiratory phonation training group (*n* = 9). This study was conducted from January 2021 in CRRC.

### Procedure

**FIGURE 1:**
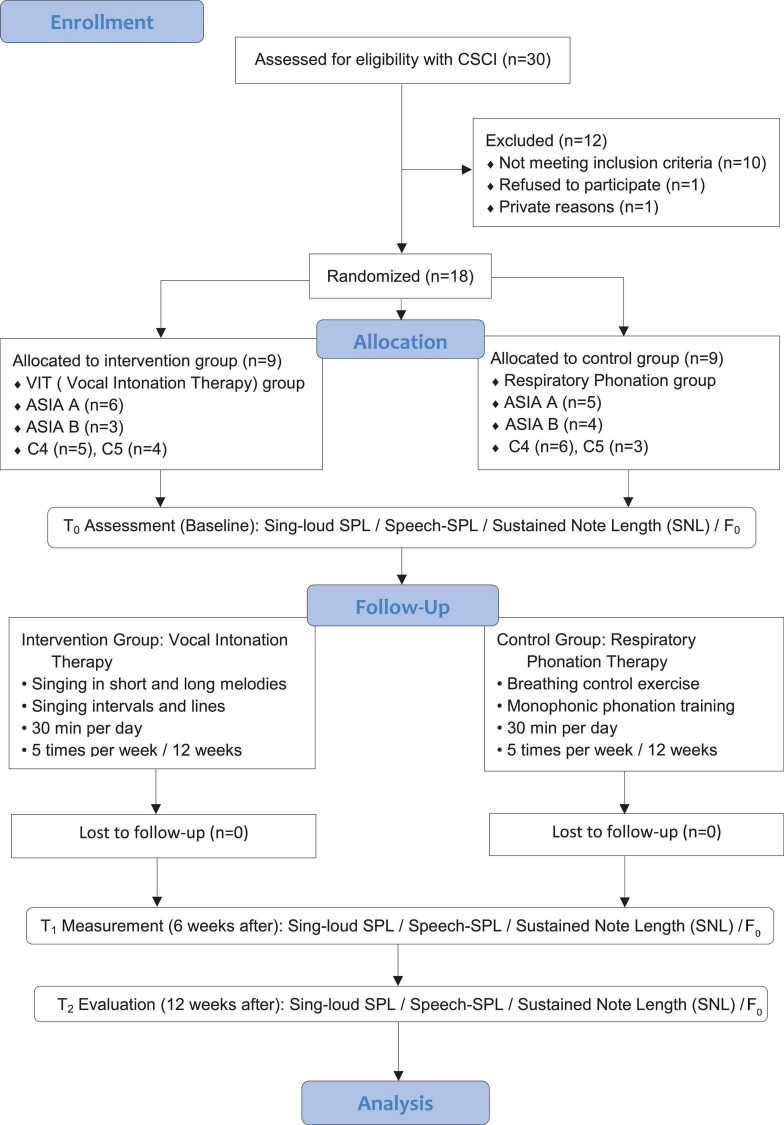
Flow diagram, consort flowchart for participants’ recruitment and allocation.

After gaining the Scientific Research Foundation of CRRC’s approval, participants diagnosed with ASIA (National Spinal Cord Injury Statistical Center [NSCISC], 2017) classification A and B with C4, C5 injuries, combined with vocal disorders, were firstly screened by clinical medical experts such as spinal cord injury specialists, spinal surgeons, and rehabilitation specialists according to the study inclusion criteria, applied for consultation and referred to the music therapy center. The music therapy researchers conducted the initial assessment and test of the vocal function of the referred patients, and the patients meeting the inclusion criteria will be included in the study to be randomly assigned, and those who fail to meet the criteria will be excluded. All patients and their families were informed and consented to this study. Participants included in the study were randomly divided into the intervention group as the vocal intonation therapy group (*n* = 9) and control group, respiratory phonation group (*n* = 9) according to the computer-generated sequence based on routine inpatient rehabilitation therapy. Patients in the intervention group were given vocal intonation therapy (VIT) by music therapists. The training process was 30 min of one-on-one VIT conducted by music therapists, 5 times per week for 12 weeks, 60 sessions in total. The control group was given respiratory phonation therapy by respiratory physiotherapists, 30 min a day, 5 times a week, for 12 weeks, 60 sessions in total. The enrollment and allocation of participants are shown in Figure 1.

Figure 1 illustrates that 30 participants were enrolled in the study, 12 participants withdrew from the study as they failed to meet the inclusion criteria (*n* = 10) dropped off the trial (*n* = 1); private reasons (*n* = 1). The intervention group was treated with VIT (total *n* = 9, ASIA A *n* = 6, ASIA B *n* = 3; C4 *n* = 5, C5 = 4); the control group underwent respiratory phonation training (total *n* = 9, ASIA A *n* = 5, ASIA B *n* = 4; C4 *n* = 6, C5 = 3). Among the 9 participants in the intervention group, all the participants were able to sing the trained items during the entirety of the study period. The data analysis included a sample of 18 CSCI patients. ASIA: American Spinal Injury Association; VIT: vocal intonation training; CSCI: cervical spinal cord injury.

### Interventions

**FIGURE 2:**
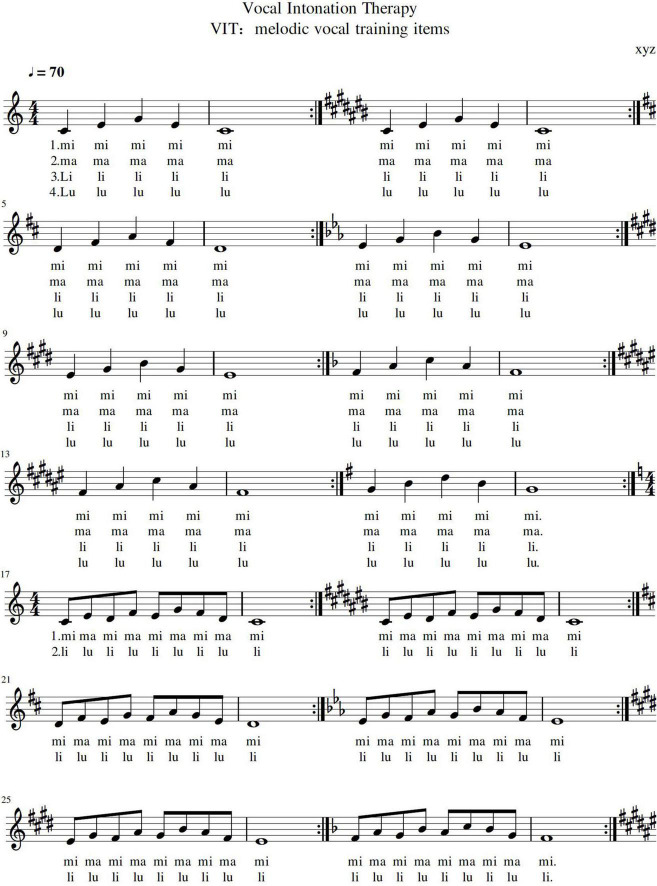
VIT items with different keys, from C to G.

Patients in the control group were given respiratory phonation training (RPT). RPT is a vocal training method whereby respiratory physiotherapists use physical therapy techniques to press the abdominal cavity with external force after the patient inhales to help them produce long sounds (Katz et al., 2022). Patients in the intervention group were treated by music therapy professionals using VIT techniques. The biggest difference between RPT and VIT is that RPT uses external force to press the patient’s abdominal cavity to guide the vocalization; while VIT is a method whereby patients make their voices autonomous under the guidance of music. In the intervention group, as well as the music therapy group, first, a vocalization assessment was performed, whereby the therapist determines the location of the difficulty based on the articulation of the patient’s voice. Following this, the preparatory exercises were then started. The specific intervention steps are: (1) Vocal warming. The therapist instructs the patient to practice monophonic vocalations starting at Andante speed (approximately 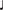 = 72), using the combined inhalation pattern of chest and abdomen, drawing respiratory to the waist and abdominal diaphragm. (2) Vocal intonation. After repeated repetitions in the previous step, the therapist instructs the patient to practice monophonic vocalizations after a quick inhalation. For example, “mi-ma-mi,” “li-lu-li” and other melody lines. The therapist uses 4/4 to complete the training of guiding the patient. During the guidance process, the patient’s vocal quality was always scrutinized. This step is repeated 20 times with different combinations of vowels, consonants, and melodies in different tones. As the final step of the intervention, therapist used a song to reinforce the previous VIT practice session (Figure 2).

**FIGURE 3:**
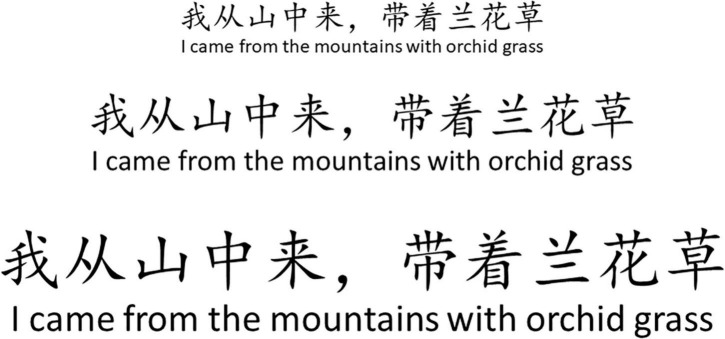
Lyric reading voice volume prompt diagram. The larger the font size is, the larger the volume required. Line 1, font number 3, low volume, about 20–30 dBA; Line 2, small 2 font, moderate volume, about 31–40 dBA; Line 3, font number 2, large volume, about 41–55 dBA.

(3) Rhythmic induced intonation. It starts with an intensive rhythm of moderate (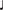 = 96), switches between word and tone combinations, exercises with a faster rhythm, and improves diaphragm jumping. Finally, the lyrics are read aloud with the volume of natural speech to normalize the vocalization function of oral communication (Figure 3). Patients in the control group were trained by respiratory physiotherapists in the bedside posture.

### Measurements

The intervention group was trained with VIT, and the control group was trained with respiratory phonation therapy at doses as previously described. The other clinical indications remained identical in both groups. Before intervention (baseline, T0), during therapy (mid-test, 6 weeks later, T1), and after therapy (evaluation, 12 weeks later, T2), using computer-aided real-time audio analyzer 2.1.6 (Adobe Systems, United States) (Reimers and Stewart, 2016) for Sing-loud Pressure Level (Sing-SPL), Speak Pressure Level (Speech-SPL), Sustained note Length (SNL), and Fundamental Frequency (F0) assessment. The measurement results in the three-time points are analyzed and compared.

#### Sing-Loud Pressure Level (Sing-SPL, Decibels)

Musical sound refers to the characteristics of hearing that can distinguish the height of musical sound, determined by the frequency of sound wave vibration, with high and low frequencies. The ratio of the effective value p(e) of the sound pressure to be measured to the reference sound pressure p(ref) is commonly used logarithmic and calculated in decibels (dBA) (Schloneger and Hunter, 2017).

#### Speech-Loud Pressure Level (Speech-SPL, Decibels)

Speech-SPL (Behrman et al., 2020) measures the effective volume of sound relative to a benchmark value with a standard sound pressure of p0 ms. Speech is the sound flow formed by the human voice language, generally expressed in decibels (dBA).

#### Sustained Note Length (SNL, s)

Musical terms are time values, and in music are the relative duration between musical tones in seconds (s) or milliseconds (ms) (Bruschettini et al., 2020).

#### Sound Frequency (F0, Hz)

In the category of the human voice, it is called the fundamental frequency, and the fundamental frequency (F0, Hz) refers to the basic sound source produced by the vibration of the vocal cords (Witold, 2020). The resulting sound unit is hertz (Hz), which is also commonly used as kilowatt-hertz (kHz) and megahertz (MHz).

### Statistical Analysis

According to the International Standard Organization (ISO), the U.S. Environmental Protection of America (EPA) (Geneid et al., 2020), the Chinese National Acoustic Environmental Quality Standard (GB 3096-2008) (Molina et al., 2021), and the Social Life Environmental Noise Standard (GB 22337-2008), office ambient noise for sound acquisition are controlled to a control standard of less than 30 dBA (Molina et al., 2021). Data was collected from both sets using a computer voice test system at three-time points before the intervention (T0, baseline), 6 weeks after (T1), and 12 weeks (T2). Formulas were used to calculate the standard deviation of the mean and normal distribution for each group, and two-way ANOVA was used to analyze differences between groups, time effects, and differences in time interactions between groups. Data from 18 patients with vocal dysfunction following CSCI were analyzed using SPSS 22.0 (SPSS Statistical Software Inc., Chicago, Illinois, United States) who completed this part of the study. Before analysis, discrete data intervals were analyzed to screen for missing and outlier values, ensure the accuracy of data entry, and determine the specific effects of interventions. Sensitive and private information was blurred out, and all statistical data were kept confidential.

## Results

### Comparison of the Overall Results of the Vocal Function Test in Two Groups

**FIGURE 4:**
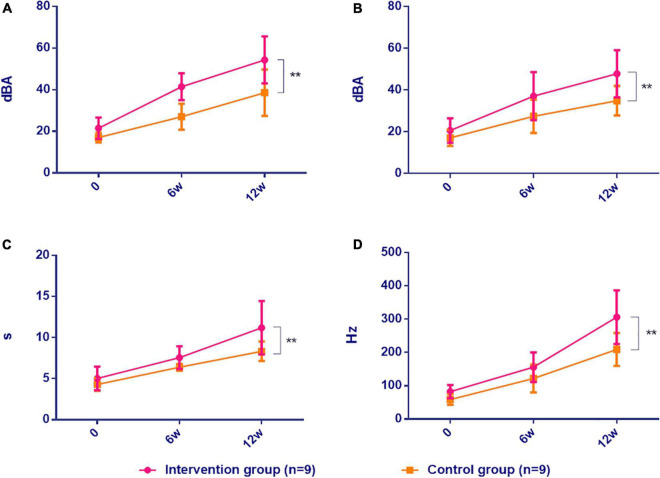
Comparison of vocal function in CSCI patients between two groups. Intervention group: vocal intonation therapy group; Control group: respiratory photation group. **(A)** Sing-SPL, **(B)** Speech-SPL, **(C)** SNL, **(D)** F_0_. Data were expressed as mean ± SD (*n* = 9) and analyzed by repeated-measures analysis of variance. *P < 0.05, **P < 0.01. 0, baseline; 6w, after 6 weeks; 12w, after 12 weeks. Sing-SPL, sing pressure level; Speech-SPL speaks pressure level; SNL sustained note level; F0, Fundamental Frequency.

Data on vocal function were collected at three-time points, before the intervention (T0, baseline), 6 weeks after (T1), and after 12 weeks (T2). These include Sing-SPL (dBA), Speech-SPL (dBA), SNL (s), and sound frequency (F0). Multivariate ANOVA was used to analyze two groups of patients T0, T1, and T2 data. The results showed that in the vocal function test, the Sing-SPL of the intervention group at the T2 time point (T2 = 54.33 ± 11.30, *p* < 0.0001), Speech-SPL (T2 = 47.83 ± 11.30, *p* = 0.0029), SNL (T2 = 11.19 ± 3.25, *p* < 0.0001), sound frequency (F0, T2 = 305.89 ± 80.39, *p* < 0.0001) possessed significant differences between groups compared with the control group. (In the analysis and comparison of the Sing-SPL, Speech-SPL, SNL, F0, the intervention group patients had a very significant inter-group difference compared with the control group and had a significant difference in time effect). The results of the statistical analysis of the two sets of data are as follows (see Table 2 and Figure 4).

**TABLE 2:** Vocal quality results in CSCI patients across the study period for the intervention and control group.

		Intervention group (*n* = 9)	Control group (*n* = 9)	*t*	*P*
		Mean ± SD	Mean ± SD		
Sing-SPL (dBA)	t_0_	21.50 ± 5.11	17.00 ± 2.40	1.226	0.0697
	t_1_	41.44 ± 6.48	27.00 ± 6.20	3.935	0.0001**^a^
	t_2_	54.33 ± 11.30	38.56 ± 11.12	4.297	0.0001**^b^
Speech-SPL (dBA)	t_0_	20.61 ± 5.92	17.11 ± 3.90	0.8819	0.2426
	t_1_	37.11 ± 11.54	27.44 ± 8.02	2.437	0.0004**^a^
	t_2_	47.83 ± 11.30	34.89 ± 7.06	3.26	0.0001**^b^
SNL (s)	t_0_	5.03 ± 1.43	4.29 ± 0.78	0.94	0.1429
	t_1_	8.16 ± 1.40	6.39 ± 0.41	1.486	0.0001**^a^
	t_2_	11.19 ± 3.25	8.33 ± 1.19	3.633	0.0001**^b^
F0 (Hz)	t_0_	82.33 ± 20.22	58.44 ± 15.56	1.077	0.05
	t_1_	155.89 ± 44.51	121.89 ± 42.14	1.531	0.0001**^a^
	t_2_	305.89 ± 80.39	208.89 ± 49.09	4.369	0.0002**^b^

Intervention group: vocal intonation therapy group; Control group: respiratory phonation group. Data were expressed as mean ± SD (n = 9), and analyzed by repeated measures analysis of variance. **P < 0.01. Superscript a represents difference factor at the same time between groups, and superscript b represents difference effect of time factor in inter-group. Sing-SPL, sing-loud sound pressure level; Speech-SPL, speech-loud sound pressure level; SNL, sustained note length; F0, sound frequency.

#### The Results Analysis of Sing-Loud Pressure Level in Two Groups

**FIGURE 5:**
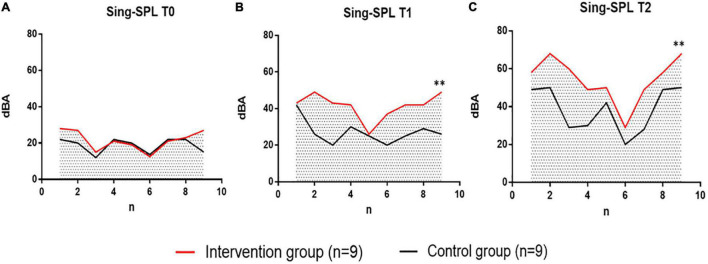
Comparison of Sing-SPL at three time points: T0, T1, and T2 between the intervention group undergoing VIT and the control group that performed respiratory phonation training. The intervention group is indicated in red. Control group, black indicates. **(A)** Individual difference distribution of Sing-SPL values at T0 time points between patients in the intervention group (*n* = 9) and patients in the control group (*n* = 9). **(B)** Individual difference distribution of Sing-SPL values at T1 time points between patients in the intervention group (*n* = 9) and patients in the control group (*n* = 9). **(C)** Individual difference distribution of Sing-SPL values at T2 time points between patients in the intervention group (*n* = 9) and patients in the control group (*n* = 9). dBA, decibel; n, the number of participants; T0, baseline; T1, after 6 weeks; T2, after 12 weeks. **p < 0.01, with significant differences between groups.

In the analysis of the results of the vocal pressure level (Sing-SPL, dBA) of patients in the intervention group and the control group, no significant differences were noted in the Sing-SPL values of the two groups of patients in the baseline test at the T0 point in time, which showed that the distribution of the two groups of patients was even and met the requirements of random control. In the T1 point-in-time (after 6 weeks) test, the Sing-SPL values of the two groups of patients began to differ, and by the T2 point-in-time (after 12 weeks), significant differences have been noted in the Sing-SPL results between the two groups. The distribution of individual values of Sing-SPL values in the two groups of patients is compared below (Figure 5).

#### The Results Analysis of Speech-SPL in Two Groups

**FIGURE 6:**
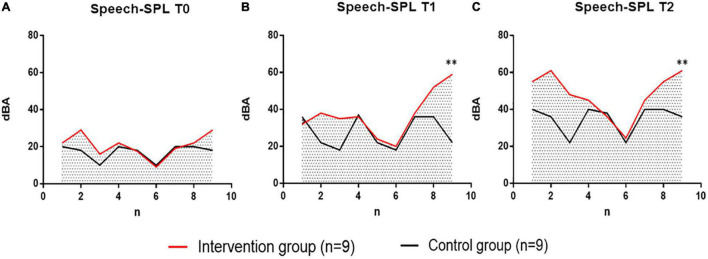
Comparison of voice pressure levels (Speech-SPL, dBA) at T0, T1, and T2 between the intervention group receiving vocal phonation therapy (VIT) and the control group receiving breathing pronunciation training. Intervention Group, intervention group, in red. The Control group is in black. **(A)** The individual difference distribution of Speech-SPL values between patients in the intervention group (*n* = 9) and patients in the control group (*n* = 9) at T0 time point. **(B)** Individual difference distribution of Speech-SPL values at T1 time between patients in the intervention group (*n* = 9) and patients in the control group (*n* = 9). **(C)** Individual difference distribution of Speech-SPL values at T2 time points between patients in the intervention group (*n* = 9) and patients in the control group (*n* = 9). dBA, decibels; N, Number of subjects; T0, baseline; T1, 6 weeks later; T2, 12 weeks later. ^**^P < 0.01, the difference between groups was extremely significant.

In the analysis of the Speech-SPL (dBA) results of the intervention group and the control group, no significant difference was noted in the Speech-SPL value of the two groups at the baseline test at T0 time point, indicating that the distribution of the two groups of patients was uniform and met the requirement of randomized control. At the T1 time point (6 weeks later), sing-SPL values began to differ between the two groups, and at the T2 time point (12 weeks later), Speech-SPL results showed significant differences between the two groups. The distribution of individual values of Speech-SPL values in the two groups was compared as follows (Figure 6).

#### Analysis of Sustained Note Length (s) Results

**FIGURE 7:**
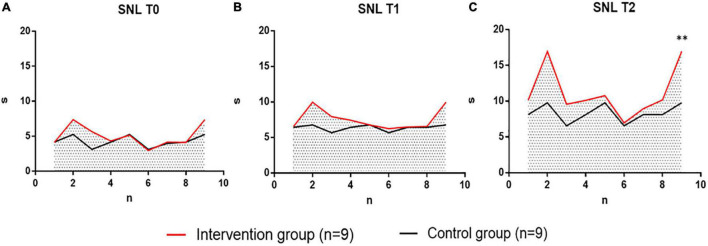
The intervention group performing vocal therapy (VIT) compared with the control group for respiratory pronunciation training at three-time points of time (Sustained Note Length, SNL, s) at T0, T1, T2. Intervention group, intervention group, indicated in red. Control group, control group, black indicates. **(A)** Individual difference distribution of SNL values at T0 time points between patients in the intervention group (*n* = 9) and patients in the control group (*n* = 9). **(B)** Individual difference distribution of SNL values at T1 time points between patients in the intervention group (*n* = 9) and patients in the control group (*n* = 9). **(C)** Individual difference distribution of SNL values at T2 time points between patients in the intervention group (*n* = 9) and patients in the control group (*n* = 9). s, Seconds; n, the number of participants; T0, baseline; T1, after 6 weeks; T2, after 12 weeks. ^**^p < 0.01, with significant differences between groups.

In the analysis of the results of Sustained Note Length (SNL) of the intervention group and the control group, no significant difference was noted in the SNL value of the two groups in the baseline test at T0 time point, indicating that the distribution of the two groups of patients is uniform and meets the requirements of randomized control. At T1 (6 weeks later), SNL values began to differ between the two groups, and at T2 (12 weeks later), SNL results showed significant differences between the two groups. The individual value distribution of SNL values in the two groups was compared as follows (Figure 7).

#### Fundamental Frequency (F0, Hz) Result Analysis

**FIGURE 8:**
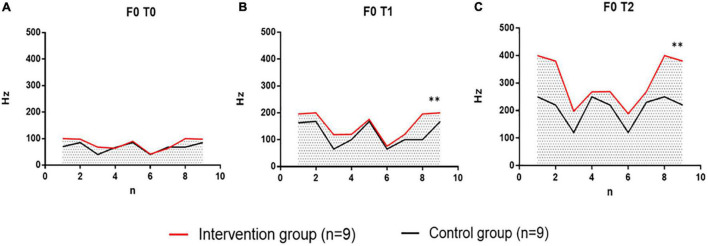
Comparison of sound frequencies (F0, Hz) at three points in time at T0, T1, and T2 in the intervention group performing vocal therapy (VIT) with the control group for respiratory pronunciation training. Intervention group, intervention group, indicated in red. Control group, control group, black indicates. **(A)** Individual difference distribution of F0 values at T0 time point between patients in the intervention group (*n* = 9) and patients in the control group (*n* = 9). **(B)** The individual difference distribution of the F0 values of the patients in the intervention group (*n* = 9) and the control group (*n* = 9) at the T1 time point. **(C)** Individual difference distribution of F0 values at T2 time points between patients in the intervention group (*n* = 9) and patients in the control group (*n* = 9). Hz, Hertz; n, the number of participants; T0, baseline; T1, after 6 weeks; T2, after 12 weeks. ^**^p < 0.01, with significant differences between groups.

In the analysis of the results of the sound frequency (F0, Hz) of the intervention group patients and the control group, no significant difference was noted in the F0 values of the two groups of patients in the baseline test at the T0 time point, indicating that the two groups of patients were evenly distributed and met the requirements of the random control. In the test at the T1 time point (after 6 weeks), the F0 values of the two groups began to differ, and by the T2 time point (after 12 weeks), there was a significant difference in F0 results between the two groups. The individual value distributions of F0 values in the two groups of patients are compared below (Figure 8).

### Spectrometry Analysis of Sing-Loud Pressure Level (Decibels) Between Two Sets of Vocal Quality

**FIGURE 9:**
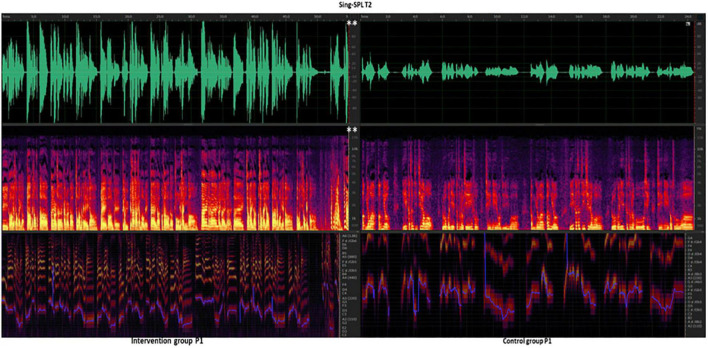
Intervention group vs. Control group at T2 time point Sing-SPL (dBA) after the 12-week intervention. P, patient. Speech-SPL T2, intervention group patients vs. control group of Speak -SPL at T2 time point of spectral analysis. Line 1, green, the waveform spectrum represents the sound decibel (dBA) value; Line 2, purple, red, and yellow, fundamental frequency (F0, Hz) values; Line 3, purple, blue, indicates pitch (reference value). White ^**^ indicates the *p* < 0.01, and the difference between groups is very significant.

In the analysis of Sing-SPL results in the intervention group patients and control group patients, it was found that there was no significant difference in Sing-SPL values between the two groups of patients in the baseline test at the T0 time point, which indicated that the two groups of patients were evenly distributed and met the requirements of randomized controls. However, after the end of all interventions, at the T2 time point (after 12 weeks), there was a significant difference in the results of Sing-SPL acoustic analysis between the two groups (Figure 9).

### Spectrometry Analysis of Two Sets of Speech-SPL (Decibels)

**FIGURE 10:**
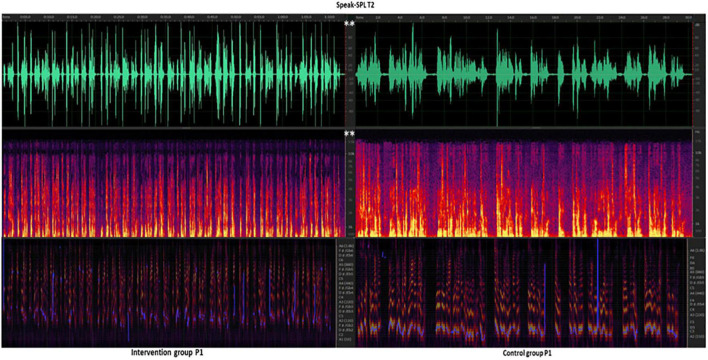
Intervention group vs. Control group at T2 time point Speech-SPL (dBA) after the 12-week intervention. P, patient. Speech-SPL T2, intervention group patients vs. control group of Speak -SPL at T2 time point of spectral analysis. Line 1, green, the waveform spectrum represents the sound decibel (dBA) value; Line 2, purple, red, and yellow, fundamental frequency (F0, Hz) values; Line 3, purple, blue, indicates pitch (reference value). White ^**^ indicates the *p* < 0.01, and the difference between groups is very significant.

In the analysis of The Speed-SPL results of patients in the intervention group and the control group, it was found that there was no significant difference in the Speed-SPL values of the two groups of patients in the baseline test at the T0 time point, which indicated that the two groups of patients were evenly distributed and met the requirements of the randomized control. However, after the end of the entire intervention, at the T2 time point (after 12 weeks), there was a significant difference in the acoustic analysis results of Speech-SPL between the two groups. Due to a large amount of data, a total of 6 patients with the most obvious changes in the intervention group and the control group were taken as an example, and the individual differences in Speech-SPL of the two groups were shown (Figure 10).

## Discussion

Impaired vocal quality is a common problem in patients with decreased respiratory function after CSCI (Qu et al., 2019). The improved voice quality related to respiratory function has become the key to enhancing the quality of rehabilitation and improving quality of life and social function in patients with CSCI (Fan et al., 2016). Subglottic respiratory volume reserve to produce vocal function, maintenance of vocal cord function, control of sound intensity (dBA), and control of subglottic pressure (SPL). Moreover, increased sound pressure provides evidence of changes in loudness for the improvement of vocal function. Most of the previous studies have focused on respiration, focusing on surface electromyography of respiratory muscles, respiratory physiological function, quality of life, and mood, while few observations have been made to improve sound quality or loudness. This study focused on the use of computer analysis-based sound spectrum analysis to observe the improvement of sound quality in CSCI patients with music therapy (Tamplin et al., 2013; Katz et al., 2022). In this study, the researchers used a meticulous and professional music intervention method to improve the sound quality of CSCI patients. 18 patients completed the 12-weeks, each participant had 60 sessions in total in the clinical trial. The improvement of musical vocal function, speech vocal function, duration, and audio frequency was observed.

### Vocal Intonation Therapy Enhances Sing-Loud Pressure Level of Cervical Spinal Cord Injury

In this study, patients with CSCI were trained to sing short melodies to long melodic vowels and consonants using VIT step by step (vowel pronunciation function and consonant pronunciation function), focusing on the output of vocal function during singing. This was manifested by an increase in Sing-SPL (T1) time point after 6 weeks compared to the baseline (T0) time point results (Figure 5), at which point there was a statistical difference between the two groups of patients receiving different training methods. Among them, the vocal pressure level of the intervention group with CSCI who received VIT was significantly higher than that of the control group who received respiratory phonation training. Their vocal function was significantly improved in the accumulation of 6 weeks. In the second course of training that followed, the vocal function of the patients in the intervention group continued to improve with the guidance of effective training methods and the increase of training time. Their vocal function improved more obviously compared to the control group after 12 weeks (Figure 9). This shows that in the cumulative time effect of the same amount of training, the vocal function of patients with CSCI in different groups has obvious differences in intervention methods: the sound pressure value of the vocal function of the patients trained in VIT has been significantly increased, and the vocal function has been significantly improved.

### Vocal Intonation Therapy Enhances Speech-SPL of Cervical Spinal Cord Injury

In this study, patients used vocalization methods to adjust vocal habits when practicing speech function. After adapting to 4 weeks of 30 min/day of training, most patients showed good behavioral changes that resulted in significant changes in speech function, which was manifested by an increase in Speech-SPL after 6 weeks (T1) compared to the baseline (T0) results. There was a statistical difference between the two groups of patients who received different training methods (Figure 6). Among them, the Speech-SPL of the intervention group CSCI patients who received VIT was significantly higher than that of the control group, which showed that after cervical spinal cord injury, patients with vocal dysfunction were trained in VIT, and their vocal function was significantly improved in the accumulation of 6 weeks. There was a more obvious improvement after 12 weeks, and the difference was more obvious (Figure 10). This shows that in the cumulative time effect of the same amount of training, the vocal function of patients with CSCI in different groups possesses obvious differences in intervention methods: the sound pressure value of a vocal function of patients trained in music therapy has been significantly increased, and the vocal function has been significantly improved.

### Vocal Intonation Therapy Significantly Increased Sustained Note Length in Patients With Cervical Spinal Cord Injury

In the SNL test, the patients in the intervention group significantly increased the duration of the continuous tone after 12 weeks, indicating that when practicing singing short melodies (up and down the third chord, Figure 2) and long melodies (three-degree melodic intervals overlapping up and down, Figure 2), long-term sound output could be performed under the support of the thoracic and abdominal resonance cavity. Patients undergo VIT therapy in conjunction with adjustments to respiratory function to support the output of vocalization. After 6 weeks of 30 min/day of training, the patients in the intervention group improved SNL in terms of the length of music and the length of speech output. The SNL in the intervention group of patients receiving VIT (T1) was significantly longer than the control group (T1) trained in respiratory pronunciation, which indicates that patients with vocal dysfunction after CSCI were trained in vocal methods, and their SNL improvement was significantly improved after 6 weeks. In the second course of training that followed, the SNL (T2) of the patients in the intervention group continued to improve with the guidance of effective training methods and the increase of training time, and the difference was more conspicuous after 12 weeks (Figure 7). This shows that in the cumulative time effect of the same amount of training, there are obvious differences in the intervention methods of SNL in different groups of CSCI patients: patients who received VIT had significantly increased their pronunciation duration and improved significantly.

### Vocal Intonation Therapy Significantly Increased F_0_ in Patients With Cervical Spinal Cord Injury

In the test of F0, the amplitude of the F0 of the patients in the intervention group increased significantly after 12 weeks, indicating that after VIT exercises and singing exercises, it was conducive to the vocal function output of high-density amplitude under the support of the thoracic and abdominal resonance cavity. Patients undergo VIT and singing training in conjunction with adjusting breathing function, while using more conserving breath to support vocal output. After adapting to 6 weeks of 30 min/day of training, the patients in the intervention group showed a change in the amplitude of sound frequency. Still, there was no significant statistical difference at that time. After continuing the training with the same amount of time accumulation for 12 weeks, the output of the sound frequency of the experimental group increased significantly. Specifically, the intervention group F0 significantly improved the accumulation of time at 12 weeks compared with the control group (Figure 8). After completing 12 weeks of therapeutic training, the F0 (T2) of the intervention group patients increased significantly with the guidance of vocal training methods and the increase in training time. This shows that in the cumulative time effect of the same amount of training, the vocal function of different groups of CSCI patients has obvious differences in the intervention method: the sound waveform amplitude of patients who received VIT vocal training and singing training was significantly enhanced, and the width of the sound frequency was significantly increased.

## Limitations

One limitation was the limited sample, as previously detailed. 12 participants dropped out of the study, which may have caused the variance in group allocation. The comparison might have been more accurate if a blank control group had been added to observe self-healing. Patients’ quality of life or patient outcome measures, or feedback from family about post-intervention functional improvement would have been a good adjunct to fit alongside the acoustic analysis. The Voice Handicap Index (VHI) may have been a useful measure to include in this study to capture any effects of the intervention on communication-related quality of life. As found in previous research using the VHI (Tamplin et al., 2013), increases in speech volume for people with CSCI make their speech more audible and intelligible to others. Besides, this study only recruited 18 patients. If larger size studies are conducted in the future, the therapeutic outcomes could be more precisely observed.

### Clinical Guidance by Vocal Intonation Therapy for the Improvement of Vocal Function

Clinicians can introduce songs gradually in order of difficulty (based on the length and pitch range of phrases). For example, a simpler song supplemental training “call-respond-chakra” singing format allows patients participating in a group practice to learn and practice together. During the first few weeks of the treatment process, the therapist can use songs with short phrases and songs with adequate breathing intervals. Actual clinical experience demonstrates that many patients with CSCI (or the general population with vocal dysfunction) initially have little confidence in their vocal function, so there is little emphasis on the quality of sound or pitch accuracy during actual VIT training. In the initial VIT singing training, the training method is mainly used to improve respiratory function and sound presentation and bring a pleasant emotional experience. Patients can sing with the accompaniment of an instrument or in karaoke style under background music recorded in the soundtrack to improve their vocal skills. According to the experience of clinical VIT plus singing training, the main recommended songs are (1) “Farewell”; (2) “Orchid Grass”; (3) “Hawthorn Tree”; (4) “Kangding Love Song”; (5) “Country Road”; (6) “The Country Road Takes Me Home”; (7) “Friendship lasts for a long time”; (8) “The Wind Blows the Wheat Waves”; (9) “Once Upon a Time”; (10) “Crooked Moon.”

## Conclusion

Vocal intonation therapy—music therapy can improve the loudness of cervical CSCI patients and provide CSCI patients with a practical, highly operable treatment that has both functional training effects and can bring a pleasant experience which can be vigorously promoted in the clinic.

## Data Availability Statement

The original contributions presented in the study are included in the article/supplementary material, further inquiries can be directed to the corresponding author/s.

## Ethics Statement

This study was reviewed and approved by the Medical Ethics Committee of China Rehabilitation Research Center (CRRC) on May 20th, 2019. Approval number 2019-83-1. Written informed consent was obtained from all participants for their participation in this study.

## Author Contributions

XZ: study design, statistical description, allocation, and statistical analysis. H-WL and D-GY: patient recruitment. Y-CS: music therapy. X-BL: project application. J-JL: study guidance. All authors contributed to the article and approved the submitted version.

## Conflict of Interest

The authors declare that the research was conducted in the absence of any commercial or financial relationships that could be construed as a potential conflict of interest.

## Publisher’s Note

All claims expressed in this article are solely those of the authors and do not necessarily represent those of their affiliated organizations, or those of the publisher, the editors and the reviewers. Any product that may be evaluated in this article, or claim that may be made by its manufacturer, is not guaranteed or endorsed by the publisher.
